# Exploring the Causal Relationship Between Telomere Biology and Alzheimer’s Disease

**DOI:** 10.1007/s12035-023-03337-4

**Published:** 2023-04-12

**Authors:** Xi-Yuen Kuan, Nurul Syahira Ahmad Fauzi, Khuen Yen Ng, Athirah Bakhtiar

**Affiliations:** School of Pharmacy, Monash University Malaysia, Jalan Lagoon Selatan, 47500 Bandar Sunway, Selangor, Malaysia

**Keywords:** Alzheimer’s disease, Telomere attrition, Shelterin complex, Oxidative stress, Aging, Neurodegeneration

## Abstract

Telomeres, also known as the “protective caps” of our chromosomes, shorten with each cell cycle due to the end replication problem. This process, termed telomere attrition, is associated with many age-related disorders, such as Alzheimer’s disease (AD). Despite the numerous studies conducted in this field, the role of telomere attrition in the onset of the disease remains unclear. To investigate the causal relationship between short telomeres and AD, this review aims to highlight the primary factors that regulate telomere length and maintain its integrity, with an additional outlook on the role of oxidative stress, which is commonly associated with aging and molecular damage. Although some findings thus far might be contradictory, telomere attrition likely plays a crucial role in the progression of AD due to its close association with oxidative stress. The currently available treatments for AD are only symptomatic without affecting the progression of the disease. The components of telomere biology discussed in this paper have previously been studied as an alternative treatment option for several diseases and have exhibited promising in vitro and in vivo results. Hence, this should provide a basis for future research to develop a potential therapeutic strategy for AD.

**Graphical Abstract:**

(Created with BioRender.com)

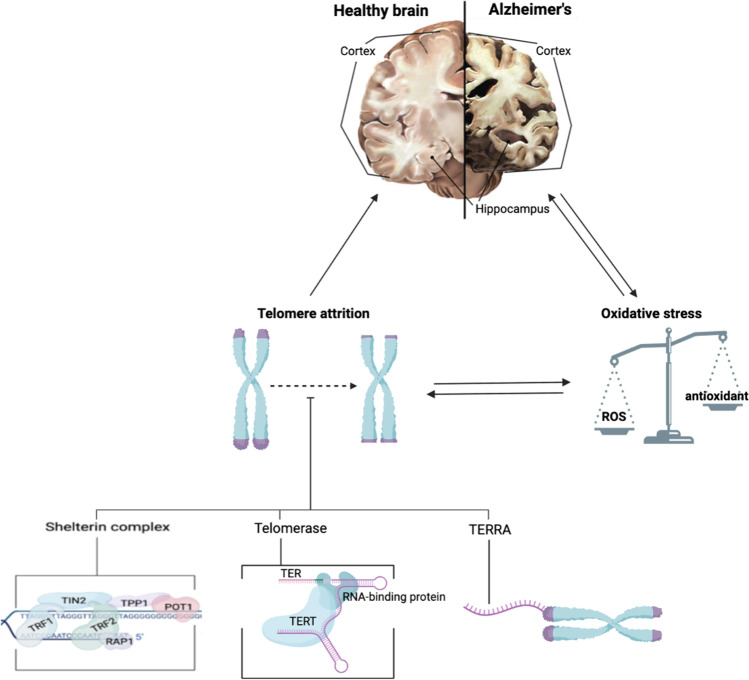

## Introduction 

Alzheimer’s disease (AD) is a progressive neurodegenerative disease and is linked with symptoms such as memory loss and diminished cognitive function. It is the most common form of dementia, contributing to approximately 60–70% of cases, and typically affects adults aged over 65 years. While it is rare, comprising around 5% of the AD population, early onsets (< 65 years old) may also occur [1-3]. Unfortunately, this disease is irreversible, and to date, there has not been any known cure for AD. In 2015, there were 46.8 million people living with dementia worldwide [4]. In the USA alone, approximately 6.07 million people were diagnosed with clinical AD as reported in the year 2020 [5]. Given the current prevalence, AD is considered to be one of the leading causes of dependence and disability [6]. Alzheimer’s Disease International reported that the estimated prevalence will continue to increase globally over the next 30 years, attributing to higher medical costs and social and informal care [4].

AD is characterized by the extracellular accumulation of protein structures called β-amyloid (Aβ) plaques and intracellular hyperphosphorylated neurofibrillary tangles (NFTs) of hyperphosphorylated or misfolded tau proteins. Over time, these histopathological features cause neuronal death and lead to shrinkages of the affected brain regions, such as the entorhinal cortex and hippocampus [7, 8]. Based on twin and family studies, it is estimated that genetic factors are involved in 80% of AD cases [9]. Depending on the age of onset and genetic predisposition, AD can be divided into 2 categories. Familial or early-onset AD (EOAD) is associated with familial mutations in the genes amyloid precursor protein (*APP*), presenilin 1 (*PSEN1*), and presenilin 2 (*PSEN2*), thereby directly affecting the production of Aβ, while more complex interactions between genetic and environmental factors contribute to the pathogenesis of sporadic and late-onset AD (LOAD). Gene polymorphisms in the apolipoprotein E (*APOE*) genes are the first to be associated with and are the strongest genetic risk factor for LOAD, affecting the disease via distinct mechanisms [9-12].

The *APP* gene is transcribed and translated into the integral membrane APP protein, which would be cleaved into different fragments by β- and γ-secretases, to finally generate the Aβ peptide [10]. The catalytic component of γ-secretases is coded by *PSEN1* or *PSEN2*, mutations in which, as seen in familial AD, would interfere with the enzymatic activity, resulting in an increased production of the longer fragment, Aβ42. This fragment is toxic and aggregates faster, as compared to Aβ fragments with shorter length, contributing to the disruption of cell membrane integrity, one of many mechanisms that could eventually lead to cell death [12-14]. A missense M233V mutation in the *PSEN1* gene was reported along with atrophy in the hippocampus-amygdala complex and cortical thinning in the temporal lobe [15]. Additionally, the *PSEN1* gene mutation is also linked with defects in the mitochondrial function, which could enhance oxidative stress [14].

One of the most invested areas of research in AD prevention is the study of telomere biology, due to its involvement in many age-related diseases [3, 16]. Meta-analysis of 13 studies has confirmed the presence of shorter telomeres in multiple somatic samples from AD, especially in leukocytes [17]. On a molecular level, AD can be characterized by several changes, such as high inflammatory cytokine levels and oxidative stress [18-21]. It has been suggested that these changes may explain the telomere shortening in peripheral leukocytes [20, 22].

While it is well-accepted that telomere length and AD share a relationship, the causal effect has yet to be elucidated. Results from previous studies are contradictory, with one reporting that mice models with short telomeres had lesser amyloid plaques compared to those with normal telomere length, slowing the progression of AD [23], while other studies suggesting that AD speeds up the shortening process [3]. By reviewing the mechanisms of telomere shortening, also known as telomere attrition, and the effect it has on human biology, this study aims to achieve a more comprehensive understanding of the causal effect between short telomeres and the onset of AD.

## Telomeres: What Are They and Why Do We Need Them?

Inside the nucleus of a human cell, deoxyribonucleic acids (DNA) are packaged into linear chromosomes, with a protective cap at the end of each arm, known as telomeres. These are double-stranded non-coding G-rich simple tandem repeats (5′-TTAGGG-3′) of lengths ranging from 2 to 20 kilobases (kb), with a 50–500 nucleotide-long G-strand overhang at the 3′ end [18, 24, 25]. Although this sequence does not code for any proteins, they are essential in the preservation of the stability of the genome by preventing the loss of critical genetic information and the unwanted recombination between chromosomes [16, 18, 19].

### Telomere Attrition

Leonard Hayflick suggested that cells have a limited proliferative potential, now coined as the Hayflick limit [26]. After a certain number of life cycles, the cell will be triggered to enter cellular senescence. It was later discovered that this may be induced by the progressive decline in telomere length [24-26]. Telomeres of critically short lengths can trigger the DNA damage response (DDR), terminating cellular replication [24, 27-29]. This poses an issue, especially in cells with high mitotic activity, such as stem cells that differentiate to, for example, replace damaged cells [18]. This indicates the importance of regulating telomere length in cell biology as the dysregulation of telomere length is often associated with numerous chronic diseases, cancers, and age-related diseases, some of which may even arise prematurely [16, 18, 19, 29].

Telomere attrition can be observed in almost every cell type, except mature red blood cells due to the absence of a nucleus and chromosomes, and is influenced by several factors, such as oxidative stress and inflammation [19]. Lifestyle factors, including diet, lack of physical activity, and smoking, have also been associated with shorter telomeres [27, 30]. Telomere attrition also occurs during cell proliferation and can be attributed to the end replication problem hypothesis, based on the mechanism of semiconservative DNA replication [16, 31]. The end replication problem hypothesized that the enzyme DNA polymerase fails to completely copy the whole DNA strand during lagging strand DNA synthesis. In linear DNA, replication of the 3′ end is carried out via the leading DNA strand synthesis, whereby it is a continuous process and moves in the direction of the replication fork. Replication of the 5′ end, however, is carried out via the lagging DNA strand synthesis, which is discontinuous and requires RNA primers. These sections are called Okazaki fragments, and two neighboring Okazaki fragments are required for a continuous daughter strand to be synthesized [31]. The process of DNA replication slows down and becomes dysregulated with age, possibly due to the relatively more loosely packed structure of the chromatins during early age [18, 32, 33].

### Short Telomere Length Is Linked with Age-Related Diseases

Due to easy accessibility, leukocytes are commonly used in many telomere length studies [34]. Short leukocyte telomere length (LTL) has been observed in several age-related diseases, including AD [32, 35]. A meta-analysis by Forero et al. involving 13 primary studies from 2003 to 2015 demonstrated consistent and significantly shorter telomeres in AD samples (*p* < 0.05). A total of 860 AD patients and 2022 controls from different continents (Europe, Asia, Australia, and North America) from this study were observed through the random-effect model, which accounts for the concept of heterogeneity, where potential differences within and between studies are taken into account. This model is more suitable for real-life scenarios, especially a biological phenomenon that is affected by many factors, from environmental to genetics. The results from this study were consistent with previous studies, with the overall standardized mean difference below zero, indicating shorter telomeres in AD patients. Subsequent sensitivity analysis further reported that the pooled result of the meta-analysis was not caused by an individual study. There was also a subgroup analysis where the reduced telomere length in leukocytes was more significant and apparent than in other cells. While this study utilized biostatistical methods to analyze available data from published studies, the study-level data could have generated biased results, although the heterogeneity was recorded at *I*^2^ = 91.8% [17].

Puhlmann et al. carried out a randomized clinical trial to investigate the effects of mental training on changes in LTL and the effects of short-term changes in LTL on cortical thickness, a measure that is more sensitive to age-related structural changes, including those seen in AD. In contrast with previous findings, the mental training in this study did not result in changes in LTL. However, a subgroup of participants who did not undergo mental training demonstrated a significant positive relationship between LTL and cortical thickness. A reduction in the cortical volume in the left praecuneus and posterior cingulate cortex was related to shorter LTL (*r* = 0.262, *p* < 0.05), whereas a thicker cortex was associated with longer LTL (*r* = 0.266, *p* < 0.05) [35].

### Mechanisms of Maintenance and Regulation of Telomere Length

#### Shelterin Complex

The telomeres resemble a double-stranded DNA break (DSB), which, if occurred in other parts of the DNA, would typically trigger the DDR. However, this is not the case for telomeres, owing to the presence of the Shelterin complex, also known as the telosome. The Shelterin complex is made up of six telomere-binding proteins (TBPs), namely telomere repeat binding factor 1 (TRF1), telomere repeat binding factor 2 (TRF2), TRF1-interacting nuclear factor (TIN2), protection of telomeres 1 (POT1), Shelterin complex subunit and telomerase recruitment factor (TPP1), and repressor-activator protein 1 (RAP1). Each of the subunits plays a specific role in regulating the dynamics of the telomere [18, 24, 36-38]. The functions of each Shelterin subunit are outlined in Table 1.

**Table 1:** The functions of the six protein subunits of the Shelterin complex. Adapted from Turner et al. [18], Shay et al. [37], and Bettin et al. [38]

Subunit	Interactions	Functions
TRF1	Binds to the double-stranded TTAGGG repeats; interacts with TIN2	Regulates telomere length
TRF2	Binds to the double-stranded TTAGGG repeats; interacts with RAP1	Regulates telomere length; stabilizes the T-loop
TIN2	Directly associated with TRF1, TRF2 & TPP1;indirectly associated with POT1	Regulates telomere length; secures TPP1 and POT1 to TRF1 and TRF2
POT1	Binds to single-stranded overhang; linked to TRF1 and TRF2 via TPP1	Regulates telomere length; prevents activation of DNA damage response
TPP1	Interacts with TIN2 and POT1	Regulates telomere length; strengthens the bond between POT1 and telomere
RAP1	Associated with TRF2	Regulates telomere length; increases affinity of TRF2 to the telomere

**Fig. 1:**
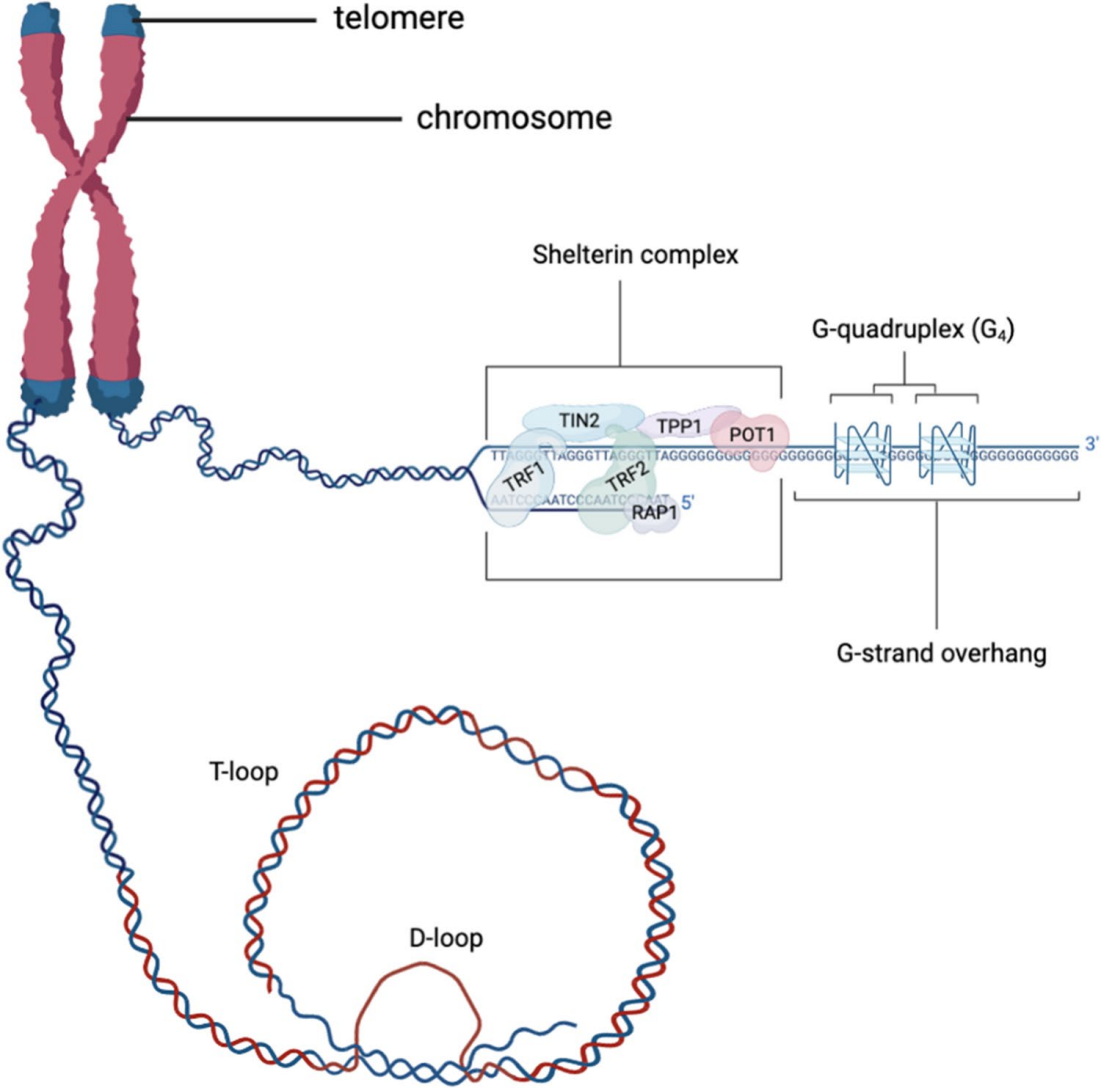
The location of the telomeres and T-/D-loop structure, the G-quadruplex (G_4_), and the Shelterin complex. The telomere consists of a double-stranded region of repeated 5′-TTAGGG-3′ DNA sequence of lengths varying from cell to cell and person to person. At the 3′ end, there is a single-stranded G-strand overhang. Telomere repeat binding factors 1 and 2 (TRF1 and TRF2, respectively) are the 2 Shelterin subunits directly bound to the telomere. A section of the telomere is folded backwards to form the T-loop. The G-strand overhang (blue line) passes the double-stranded region of the telomeric DNA to form the D-loop (red line). (Created with BioRender.com)

The Shelterin complex protects telomeres by organizing them into a T-loop, a lasso-like structure that shields the telomeres from recognition by the DDR [24, 37, 39]. The disruption of the T-loop may also affect gene expression [37]. As seen in Fig. 1, the G-strand overhang is capable of folding into G-quadruplex (G_4_) structures, a secondary structure consisting of four guanines connected via Hoogsteen hydrogen bonds [40, 41]. The G-strand overhang can also loop back into the double-stranded DNA to form the T-loop [16, 40]. One other structure that helps stabilize the T-loop is the displacement loop (D-loop), which is formed by the single-stranded G-rich overhang invading into the double-stranded telomeric DNA and displacing one of the strands [25].

The presence of TRF2 is necessary for the formation of the T-loop. The subunit wraps ~ 90 base pairs of DNA around its homology domain (TRFH) to form the loop, in a process involving lysine and arginine residues on the subunit. A mutation in these residues, termed Top-less, will result in the disruption of the loop and the subsequent lack of protection against the ataxia telangiectasia mutated (ATM)-dependent DDR pathway [42]. Upon activation by DSBs, this pathway triggers a series of phosphorylations of several substrates that mediate DNA repair and cell cycle, which could cause telomere fusions [24]. Timashev et al. demonstrated that in the absence of the expressions of TRF1, TIN2, TPP1, POT1, and RAP1, mouse embryo fibroblasts expressing only TRF2 exhibited an average number of T-loops. The cells that did not express TRF2 reported low levels of T-loop formation [43]. While this highlights the primary importance of TRF2 out of all the Shelterin subunits, the maintenance of the T-loop structure is also dependent on the collaborative effort with other subunits, as demonstrated by Mahady et al. The study revealed significant telomere attrition in the precuneus, the area that is involved in memory deficits in the prodromal stage of AD. The levels of TIN2, POT1, TRF1, and TRF2 were reported to be significantly reduced in mild cognitive impairment (MCI) samples, and even more so in AD samples. In addition, these samples also exhibited significant telomere attrition [44]. This finding deduced that the dysregulation of the Shelterin is involved in telomere attrition.

TRF2 recruits subunit RAP1 to stabilize and enhance its binding to the telomeric DNA. RAP1 is a highly conserved Shelterin subunit, involved in the protection of telomeres. RAP1 consists of 3 domains, namely the BRCA1 C-terminus (BRCT), myeloblastosis (Myb), and RAP1 C-terminal (RCT) domains. The absence of the BRCT domain can result in the loss of telomere length heterogeneity [45]. RAP1 also assists TRF2 to protect the T-loop, which preserves the integrity of the genome and resembles the intermediary structure of homologous recombination [46]. The absence of the BRCT domain makes the telomeres more prone to recombination via homology-directed repair (HDR), resulting in telomere resection and fusion, and ultimately telomere loss [47]. While RAP1 prevents the activation of non-homologous end joining (NHEJ), this DDR pathway is not necessarily activated in its absence. It was later suggested that this function primarily acts as a failsafe mechanism, for example, when telomeres are too short for T-loop formation [48]. Lototska et al. demonstrated that by treating cells that have critically short telomeres with RAP1, it was possible to reverse senescence [49]. So far, over 50 genetic mutations in RAP1 have been reported in several diseases, mostly cancers [45].

TRF1 is structurally similar to TRF2 as it possesses the same domain for direct binding to the telomeric sequence. Functionally, TRF1 is mainly involved in regulating telomere lengths and promoting its replication [48]. Besides that, it was demonstrated that dysregulated TRF2 can stimulate telomeric invasion, a process that would ultimately result in telomere loss, and that TRF1 can inhibit this process [50]. Wu et al. reported that TRF1 levels in peripheral blood leukocytes decreased in AD patients expressing higher Aβ42 but increased in those expressing higher tau protein, and vice versa for TRF2. Hence, the pathologies of AD can influence the expression of the Shelterin subunits [51].

#### Telomerase

Telomere attrition is not a permanent phenomenon as it can be restored by the enzyme telomerase. As a reverse transcriptase, telomerase works by adding TTAGGG sequences to the end of telomeres, hence elongating them. Telomerase comprises the catalytic telomere reverse transcriptase (TERT), telomerase RNA (TER), and accessory proteins such as NOP10, NHP2, dyskerin, and GAR1 [3, 52, 53]. Telomerases are found abundantly in cells with high proliferative capacities, such as stem cells and cancer cells [19]. Telomerase activity is high during early human development but is greatly downregulated in most adult somatic cells [37, 40], likely due to several silencing mechanisms that have yet to be elucidated [54].

The expression of TERT correlates with telomerase activity [55]. However, it can also regulate transcription via its non-catalytic interaction with other enzymes, upregulating the expression of neurotrophic factors, and thus preserving cognitive function [56, 57]. The absence of TERT is reflected in the reduction of doublecortin, a marker of neurogenesis, which is a repair mechanism that can mitigate the effects of neurodegeneration in AD [58]. TERT is also found to have protective effects against Aβ-induced neurotoxicity [56, 57].

Recently, a novel gene transcription regulatory mechanism was discovered. Described as the telomere position effect over long distances (TPE-OLD), Kim et al. reported that *TERT* expression is repressed in cells with long telomeres, but is upregulated as the telomeres shorten [54]. Essentially, TPE-OLD is a mechanism whereby telomeres of sufficient length can interact with *cis*-genomic regions up to 10 Mb away, forming a telomere loop, hence silencing that targeted gene. However, when telomeres shorten, as seen with aging, this mechanism gets compromised, causing the telomere to disengage with the sequence and resulting in the expression of that particular gene [59, 60]. While the regulation of *TERT* via this method appears to be protective against replicative senescence, a few other genes have also been found to be regulated the same way and it does not necessarily have a positive effect [61]. For instance, TPE-OLD has been associated with facioscapulohumeral muscular dystrophy, where cells with shorter telomeres resulted in the upregulated expression of the disease-causing gene *DUX4* [62]. However, it is still unsure whether AD genes are also regulated via this mechanism.

Telomerases are recruited to the telomere by the Shelterin subunit TPP1 [38, 63]. In addition to this, TPP1 also enhances the already-high binding affinity of POT1 to the single-stranded telomeric DNA by holding the subunit close to the telomere [64]. POT1 alone, when bound to the telomeric DNA, inhibits telomere elongation by preventing the binding of telomerase. However, when TPP1 and POT1 bind the telomeric DNA together, telomerase is anchored to the telomere, hence allowing telomere elongation to occur [64-66].

The TPP1-POT1 complex interacts in a very precise manner with telomerases to promote a process known as repeat addition processivity (RAP), where telomerase can synthesize more repeats [63, 65, 67]. Enhanced RAP by this interaction can occur via two mechanisms: by inhibiting the dissociation of the enzyme or by assisting the translocation of the template strand [66, 67]. Hwang et al. proposed that the latter mechanism could be due to the dynamic sliding of TPP1-POT1, whereby it binds to the overhang of one oligonucleotide/oligosaccharide binding (OB)-fold (that interacts with one repeat sequence) at a time, unfolding the G_4_ structure in a stepwise manner. However, Zhang et al. reported that a mutation of the residue Serine 111 (S111) found on the OB-fold of TPP1 could decrease telomerase activity. This mutation prevents the phosphorylation of the residue, which is essential in the recruitment of telomerase, thus resulting in telomere attrition [68]. However, the S111 mutation has not been directly associated with any neurodegenerative diseases thus far. Nevertheless, this provides a potential point of intervention that can be explored further for therapeutic purposes.

#### Telomeric Repeat-Containing RNA (TERRA)

While telomeres have always been thought to be transcriptionally silent, recent studies have discovered that TERRA is formed as a result of transcribing the telomeric sequence with assistance from the Shelterin subunit TRF1. Similar to telomeres, TERRA is a long and repeated sequence that does not code for any proteins. As it is an RNA, TERRA is composed of UUAGGG, with uracil (U) in place of thymine [39].

The TERRA promoters are found in the subtelomeric region. The subtelomeres can exist in one of two forms, one with a CpG island, and the other without. The cytosines are the main target of DNA methylation. DNA methylation at the promoter region typically suppresses gene expression by preventing the binding of transcription factors, and it is positively correlated with telomere length [69]. An increase in TERRA expression was observed in cancer cells that were hypomethylated [70].

The effect of enhanced TERRA expression on telomere length regulation was demonstrated in a study involving cells from a rare autosomal recessive disease called immunodeficiency, centromeric region instability, and facial anomalies (ICF) syndrome, where the cells express shorter lengths of telomeres. The study revealed that high levels of TERRA resulted in the formation of DNA-RNA hybrids, whereby the G-rich TERRA would bind with its C-rich DNA template strand via complementary base pairing [71]. This hybrid formation disrupts the genome integrity by causing fork stalling, which would result in DSBs and potentially the loss of telomere length without triggering DDR.

Cusanelli et al. reported that the expression of TERRA is upregulated at short telomeres [72]. RAP1 suppresses the expression of TERRA by interacting with inhibitory proteins such as Sir and Rif. With short telomeres containing fewer RAP1 binding sites, the repressive effect of RAP1 on the expression of TERRA is reduced, resulting in increased TERRA levels. On the contrary, the restoration of telomere length would re-establish the expression of TERRA [72]. Upon TERRA transcription, the TERRA focus is formed. In *Saccharomyces cerevisiae* cells, during the S phase of the cell cycle, TERRA interacts with the components of telomerase to form the telomerase recruitment clusters (T-recs). In the late S phase, these T-recs would be recruited to their telomere of origin. The mechanism by which they assemble is still unknown. However, the formation of T-recs reflects the activity of telomerase as it is dependent on the factors that are involved in telomerase-dependent elongation [72]. The findings from this study implied the involvement of TERRA in the activity of telomerase at the telomeres.

TERRA is also expressed in human cells and can interact with human telomerase RNA and human TERT (hTERT) in vivo [72]. A more recent discovery found a direct inhibitory effect of TERRA on telomerase [38, 40]. This is likely dependent on the interaction between the G-rich sequence of TERRA and the template region of the telomerase. Additionally, TERRA can fold into a G_4_ structure that allows it to bind to TRF2 which affects the structure of the T-loop [39, 73].

Telomeres are originally in a heterochromatin form and are seen with high levels of CpG methylation, along with histone 3 lysine 9 trimethylation (H3K9me3) and the trimethylation of the histone 4 at Lys20 (H4K20me3) [39]. These are thought to be negative regulators of the elongation of telomeres [74]. Depending on the type of markers or enzymes present in the cellular environment, TERRA can regulate the telomere length by altering the chromatin structure via stabilizing the heterochromatin structure or promoting the transformation to a euchromatin structure. TERRA can stabilize the heterochromatin form by interacting with several proteins, including heterochromatin protein 1, methyl CpG binding protein, and origin recognition/replication complexes [38].

## Oxidative Stress

Oxidative stress is the result of an imbalance in the redox equilibrium, described as an excessive synthesis of reactive oxygen species (ROS) with insufficient production of antioxidants [75, 76]. It can damage plasma and mitochondrial membranes via lipid peroxidation, and cause irreversible modification to structural and enzymatic proteins via oxidation [77]. Additionally, ROS, such as hydrogen peroxide (H_2_O_2_), superoxide, and nitric acid, can interfere with and cause damage to the genomic DNA, triggering the DDR. While this is essential at certain sections of the DNA strand, DDR can disrupt the integrity and stability of the genome if targeted at the telomere. This is because the DDR recognizes telomeres as a single-stranded break (SSBs), and any attempt to fix it will eventually make it double-stranded. As the single-stranded characteristic is necessary to form the T-loop, this will no longer be possible [37]. Consequently, the cells undergo cellular senescence and even apoptosis. This can be observed in the pathogenesis of many health conditions, including cardiovascular diseases, carcinogenesis, and neurodegenerative diseases [76, 78, 79].

ROS are reactive molecules that, if accumulated, can cause a cascade of chemical reactions and cause structural changes. At moderate levels, ROS molecules play an important role that is beneficial to the health of the organism [76]. For instance, free radicals synthesized and stored in phagocytes can be released upon invasion of pathogens to destroy foreign bodies. ROS also function as signaling molecules that are important in regulating physiological events such as growth factor signaling, cellular proliferation, and differentiation. This usually involves the interaction of ROS with the cysteine residues on proteins. For example, H_2_O_2_ can oxidize cysteine on a membrane protein, which would result in a conformational change, thus increasing membrane permeability. Subsequently, this facilitates transcription, phosphorylation, and other physiological events [80]. The role of ROS in the regulation of cellular proliferation can be observed in several cancer-related pathways, such as phosphoinositide 3-kinase (PI3K) and nuclear factor κ light-chain-enhancer of activated B cells (NF-κB) [81].

**Fig. 2:**
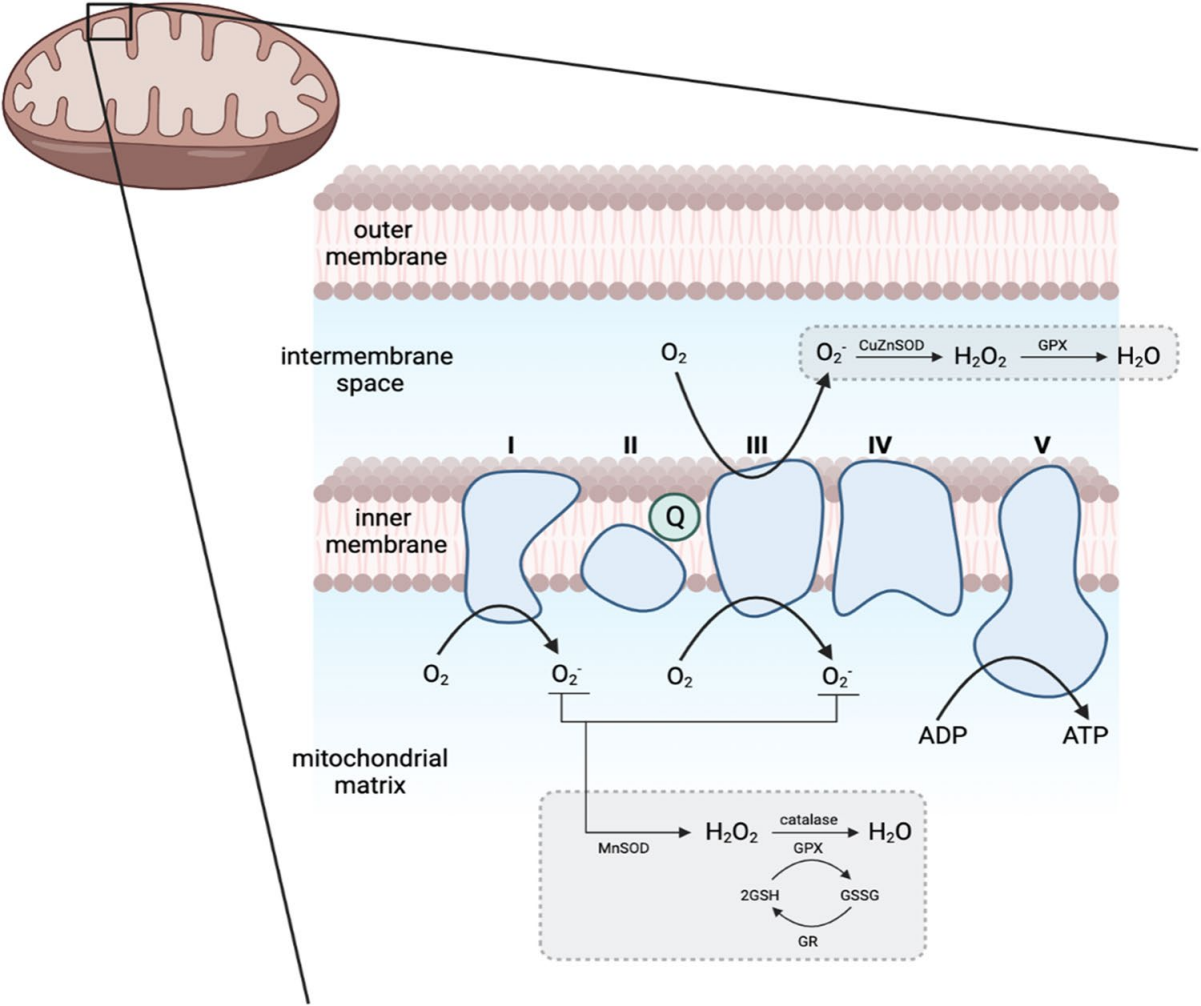
Formation of ROS in the matrix of the mitochondria. The turquoise structures represent the complexes (I–IV) of the electron transport chain. During aerobic respiration, complexes I and III convert oxygen molecules (O_2_) to superoxide anion (O_2_^−^), becoming the major sources of ROS. O_2_.^−^ can be converted to water (H_2_O) with hydrogen peroxide (H_2_O_2_) as an intermediary product. Glutathione (GSH) acts as an antioxidant in this process. Adapted from Tönnies et al. [79]. (Created with BioRender.com)

Mitochondria are the main source of ROS, which are formed as a by-product of oxidative phosphorylation that takes place at the electron transport chain (ETC) in the inner mitochondrial membrane (Fig. 2). The ETC essentially recycles electrons from electron donors, in the process reducing oxygen molecules (O_2_) into superoxide anion (O_2_^−^), which can spontaneously undergo dismutation into hydrogen peroxide (H_2_O_2_), both of which become the main sources of ROS [82]. Under normal circumstances, the mitochondria can regulate ROS levels. However, with age, mitochondrial dysfunction becomes more common, disrupting this equilibrium [8, 75, 76].

### How Is Oxidative Stress Associated with Telomere Attrition and AD?

The majority of the DNA damage in the brain is likely caused by oxidative stress due to the great demand for oxygen and the high metabolic rate of brain cells [77, 83, 84]. The markers of oxidative stress, such as high concentration of oxidized proteins, and oxidative modifications in mitochondrial and nuclear DNA, have also been documented in post-mortem brain tissues, especially from regions involved in AD, such as the hippocampus and parietal cortex [79, 85].

Wahlster et al. demonstrated that in sporadic AD, aging-related accumulation of ROS causes conformational changes in the catalytic subunit of γ-secretase in neurons, resulting in an increased production of Aβ42, the same way familial AD-associated mutations in *PSEN1* would affect the Aβ production [86]. The deleterious effects of aggregated Aβ on telomere maintenance were demonstrated in a study by Qin et al. wherein pheochromocytoma (PC12) tumor cells were treated to overexpress Aβ42 [87]. The phosphorylation of H2AX, a DSB marker, was observed 12 h post-transfection of a plasmid containing coding for Aβ42 gene, demonstrating the ability of Aβ42 to induce DNA damage. Further investigation revealed that Aβ42 was a telomere target peptide that is capable of dissociating POT1 and TRF2 from the telomere. Consequently, there was an increase in end-to-end joining following the dysregulation of telomere capping. Additionally, Aβ42-induced oxidative stress reduced TERT expression and telomerase activity. This interferes with the maintenance of telomere length, eventually leading to cellular senescence, which can be alleviated by the re-introduction of POT1 and TRF2 [87]. However, Aβ is typically present in the extracellular environment, and the mechanism of intracellular production and its possible localization were not taken into account in this study. Hence, this can only serve as a reference as to how Aβ would affect the maintenance of telomere length, and not as a conclusive statement that it occurs in AD.

The phosphorylation of tau is mediated by the balance between the action of protein kinases and phosphatases. Disruption of this balance results in its hyperphosphorylation, which ultimately destabilizes the neuronal cytoskeleton as it loses its ability to bind with the microtubules, and impairs synaptic function, as observed in AD [88, 89]. Tau phosphorylation is also modulated by mitochondrial oxidative stress [90]. In a study attempting to demonstrate the risk of excessive alcohol consumption on developing dementia, Li et al. reported that acetaldehyde-treated human neuroblastoma cells had an increased production of phosphorylated tau via the elevated ROS levels and activation of several protein kinases, such as p38 mitogen-activated protein kinase (MAPK) and c-Jun N-terminal kinase (JNK) [90]. Additionally, telomere attrition is also known to activate the ATM pathway, which in turn also mediates the activation of p38 [91]. Abnormal tau phosphorylation can also contribute to mitochondrial damage and increased oxidative stress in neurons [92]. Under healthy conditions, the balance between mitochondrial fusion and fission is well-maintained. In the presence of elevated phosphorylated tau, however, this balance is disrupted, subsequently affecting the distribution of mitochondria to neurites and leading to axonal and synaptic degradation [88, 93]. Despite the lack of concrete evidence showing the direct link between telomere attrition and hyperphosphorylated tau, it is possible that a crosstalk may occur via oxidative stress.

Among all the nucleic bases, guanine has the lowest redox potential. Thus, the G-rich sequence of the telomere is the most susceptible to oxidative damage [18, 94, 95]. Coluzzi et al. demonstrated that there was a more significant effect on telomeric DNA following treatment with two doses of H_2_O_2_ than on genomic DNA. The difference between the extent of genomic and telomeric DNA damage is also suggestive of a less efficient repair system in the telomeric region compared to the rest of the genome [94]. The compound 8-oxoguanine (8-oxoG) is formed as a result of the oxidation of guanine and is a commonly used marker in the detection of oxidative damage in numerous diseases. Both 8-oxoG and its nucleotide form, 8-hydroxydeoxyguanosine (8-OHdG), are highly accumulated in the brain and peripheral lymphocytes of AD patients [83, 95-97]. It has been reported that 8-oxoG is highly mutagenic and cytotoxic as it can bind to adenine and its complementary base pair cytosine [95, 97].

The lesions of 8-oxoG are repaired by the base excision repair (BER) mechanism, which is initiated by DNA glycosylases upon recognizing the oxidized base. These enzymes work by removing mismatched base pairs in the DNA strand and adding the correct nucleotide. Examples of DNA glycosylases include OGG1, which excises the 8-oxoG opposite a cytosine, and MUTYH genes, which removes the adenine mispaired with 8-oxoG [20, 83, 84, 98]. If 8-oxoG lesions are not repaired correctly, it may result in SSBs or DSBs [94]. In addition, the enzyme human MutT homolog 1 (MTH1) also helps to hydrolyze the different forms of 8-oxoG, preventing its incorporation into the DNA [99]. However, MTH1 and OGG1 are significantly reduced in sporadic AD cases. Oka et al. revealed that this, accompanied by the subsequent accumulation of 8-oxoG in the hippocampal and cortical neurons, can also trigger microglial activation and neuronal loss, contributing to the pathogenesis of AD [97]. It was also reported that the OGG1 mRNA transcripts were reduced in MCI (due to AD pathology) and AD patients, and also patients expressing normal (which includes MCI patients with no AD pathology and patients with subjective cognitive impairment) and abnormal levels of Aβ42 and tau, as compared to healthy controls. This signifies that the alterations in OGG1 transcript levels may be independent of AD pathologies and that it may be an event preceding the onset of the disease, as a difference in OGG1 transcript level, as compared to healthy controls, was also observed in patients with no AD pathologies [100].

MUTYH is highly expressed in the striatum, the part of the brain involved in motor and cognitive functions. MUTYH repairs the lesions of 8-oxoG, but in the process, may also trigger neurodegeneration. Sheng et al. demonstrated that OGG1-knockout mice expressing MUTYH presented an increased neuronal and microglial cell death in the striatum as a result of the accumulation of 8-oxoG. It was proposed that this may likely cause high oxidative stress-induced neurotoxicity [101]. Mizuno et al. also reported that MUTYH, found in most of the hippocampal neurons and glia regardless of the AD status, contributed to memory impairment via microgliosis and accelerating neuronal dysfunction [98]. The adenine excision initiated by MUTYH induces the accumulation of SSBs in the DNA, which triggers different cell death pathways. For instance, since mitochondria are the primary source of oxidative stress, 8-oxoG significantly accumulates in the mitochondrial DNA, initiating calpain-dependent neuronal loss. Meanwhile, 8-oxoG accumulation in neuronal DNA triggers the PARP-AIF pathway that results in microgliosis [101].

Furthermore, 8-oxoG also affects telomerase activity and the binding of Shelterin subunits to the telomere sequence, consequently disrupting telomere elongation [94]. As discussed earlier, Shelterin subunits have crucial roles in the maintenance of the telomere and require specific telomeric interactions to carry out their functions. Opresko et al. reported that TRF1/2-bound substrates were reduced by at least 50% due to the presence of an 8-oxoG lesion, caused by the failure of the BER mechanism [102].

**Fig. 3:**
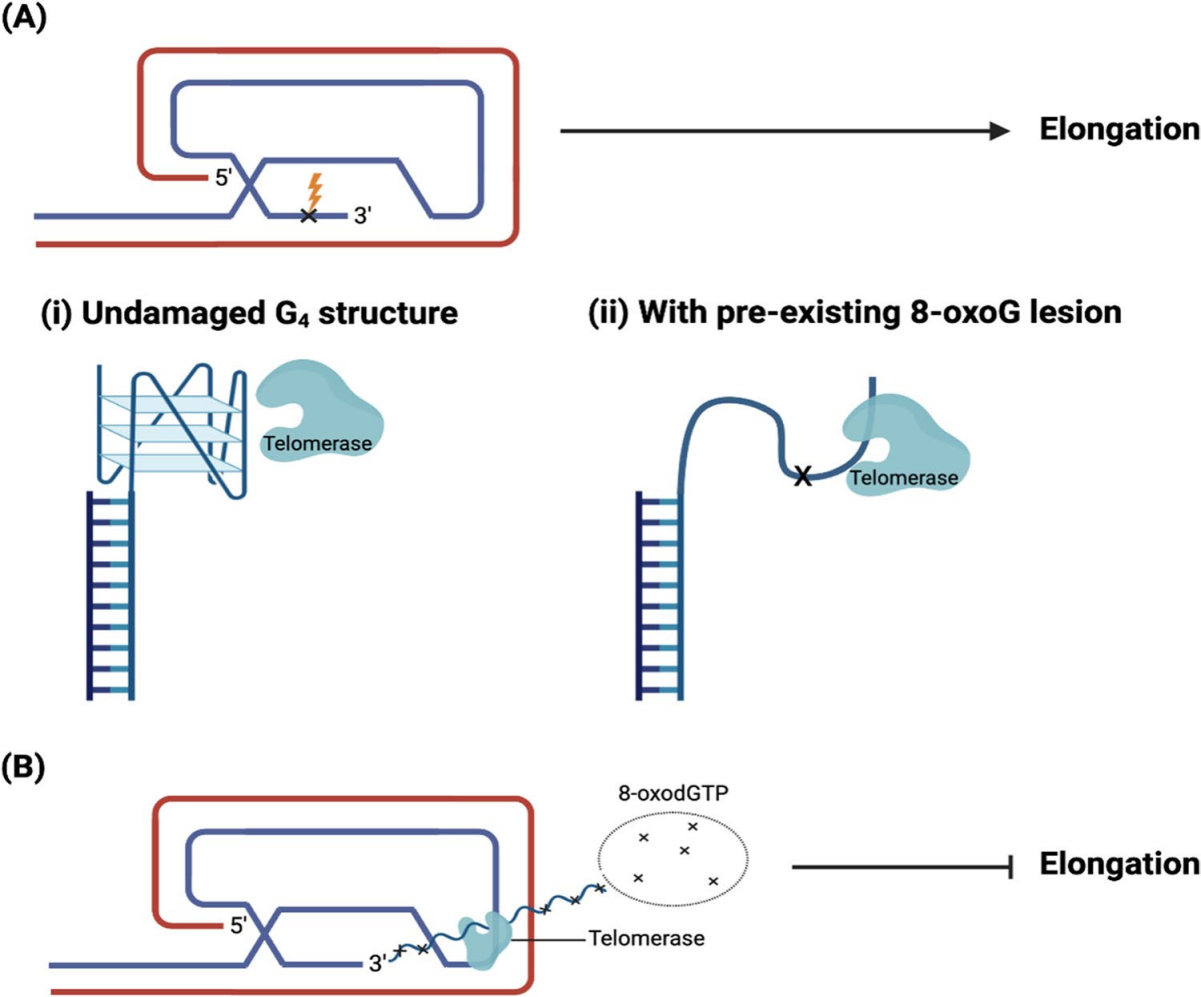
The model of the dual role of 8-oxoG in inhibiting or promoting telomerase activity. **A** Pre-existing 8-oxoG in the loop that arises from direct DNA damage (ii) disrupts the G_4_ structure (undamaged G_4_ structure shown in (i)), hence allowing telomerase to access, in turn promoting elongation. **B** 8-oxoG in a pool of oxidized dNTPs exists as 8-oxodGTP. Its insertion into telomeric DNA by telomerase prevents elongation of the telomeres and hence, synthesis is terminated. Adapted from Fouquerel et al. [103] and Lee et al. [104]. (Created with BioRender.com)

Fouquerel et al. reported a dual role of the 8-oxoG (Fig. 3), whereby its cellular environment determines whether it enhances or inhibits the activity of telomerase. This study demonstrated that, other than the direct oxidation of guanine in the overhang, the incorporation of 8-oxodGTP into telomeric DNA also produces 8-oxoG [103]. Pre-existing 8-oxoG in the G-strand overhang would increase the accessibility of telomerase by disrupting the Hoogsteen hydrogen bond, hence destabilizing the G_4_ structure of the overhang [41, 103, 104]. When 8-oxoG is present within an oxidized deoxynucleoside triphosphate (dNTP) pool in the form of 8-oxodGTP, its incorporation into the telomere prevents the binding of catalytic enzymes, hence preventing telomere elongation and possibly triggering cell senescence [103]. Nevertheless, persistent 8-oxoG formation at selective telomeric regions can directly affect genomic instability by inducing chromatin bridges, micronuclei formation, and chromosome end fusions, and this is exacerbated in the absence of OGG1 [105]. However, OGG1 can only work if 8-oxoG is paired, and not when it is present in the single-stranded overhang or within the G_4_. A 4-TTAGGG repeat substrate modified to contain 8-oxoG reported an improved telomerase activity as compared to the unmodified 4-TTAGGG repeat substrate. The modified substrate was more loosely folded as compared to the unmodified substrate, thus, increasing the accessibility of telomerase [103].

OGG1 is largely expressed in the nervous system [83, 84, 96]. The removal of the mispaired base by this enzyme forms an abasic (AP) site. AP-endonuclease 1 (APE1) will then hydrolytically cleave the phosphodiester backbone at the site to allow for end-processing and DNA synthesis by polymerase β (Polβ). Finally, the strands are joined together by DNA ligase [100]. If glycosylase activity is compromised, 8-oxodG cannot be effectively removed from the genome and a sequence of errors follows. For example, mitochondrial polymerase γ (polγ) and nuclear polymerase δ (polδ) will pair 8-oxodG with an adenine, both of which result in a point mutation in the genome. Additionally, other enzymes, such as RNA polymerase, can easily bypass this lesion. In some cases, this can result in a miscoded or prematurely terminated mRNA. This highlights the importance of a fully functioning OGG1 in the defense against mutagenic oxidative damage and protection of the genome [106].

The activity of BER is influenced by single nucleotide polymorphisms (SNPs) in DNA repair genes. Currently, over 20 polymorphisms in *OGG1* have been identified and 13 of which are associated with diseases [107]. In addition, BER may have been downregulated in some AD cases. Mao et al. identified two polymorphisms in the *OGG1* gene in two out of 14 AD brains: A53T and A288V [108]. Jacob et al. demonstrated that these two polymorphisms disrupted the interaction between OGG1 and the other members of the BER pathway. The catalytic activity of the enzyme was also significantly reduced. Ultimately, these SNPs reduced the cell survival rates [107].

More recently, another SNP also started garnering more interest as it can also reduce OGG1 activity. Baptiste et al. demonstrated that *OGG1* with the S326C polymorphism was less efficient in removing 8-oxodG, as compared to normal *OGG1*. In time, 8-oxodG can accumulate and cause further damage [106]. Dinçer et al. reported significantly higher oxidative DNA damage and 8-hydroxydeoxyguanosine (8-OHdG) levels in AD patients with this SNP, as compared to those without [83]. The S326C SNP is influenced by smoking and environmental factors, such as pollution, and it is more commonly seen in the Asian population [106]. Furthermore, it was recently discovered that an age-dependent increase in oxidative stress leads to telomere attrition in the post-mitotic stage of induced pluripotent stem cell (iPSC)-derived motor neurons expressing the C9ORF72 mutation, which is commonly seen in two incurable neurodegenerative diseases, namely amyotrophic lateral sclerosis (ALS) and frontotemporal dementia (FTD). While there were no changes in the protein expressions of TRF2 and POT1 in neuroepithelial cells and motor neuron progenitors, these protein levels were reduced in two-month-old post-mitotic motor neurons bearing the C9ORF72 mutation, which caused the exposed ends of the chromosomes to trigger the DDR [109].

### Protective Effects Against Oxidative Stress

The neuropathological hallmarks of AD can cause defects in some mitochondrial components in transgenic mouse models [110]. For instance, Aβ was found to cause deficits in the Complex IV of the ETC in the mitochondrial matrix, whereas hyperphosphorylated tau selectively impairs Complex I [110]. These complexes are involved in the recycling of electrons, with some amount of ROS being generated as a by-product in the process. While this highlights the involvement of mitochondria in the pathogenesis of AD, it remains to be elucidated as to whether mitochondrial dysfunction is a cause or consequence of the presence of Aβ and tau, and if it has a direct role in the onset and progression of the disease.

TERT was found to have protective effects against the pathology of AD, particularly the tau protein in the hippocampal region. The presence of TERT was necessary to limit the levels of ROS and prevent oxidative damage in neurons. In vitro studies demonstrated that in the event of tau-induced oxidative stress, TERT translocates from the nucleus to neuronal mitochondria and was able to restore the activity of Complex I, thereby lowering the ROS levels and subsequently preventing DNA damage [8].

A more recent study found that telomerase protects mitochondrial DNA against oxidative stress by enhancing the antioxidant defense mechanism. Instead of directly interacting with the BER pathway, telomerase promotes the production of the antioxidant enzyme manganese superoxide dismutase (MnSOD), via an interaction with the transcription factor forkhead box protein O3. Martens et al. observed a significant increase in MnSOD levels following the overexpression of hTERT in human fibroblasts that were treated to undergo oxidative stress. Consequently, oxidative stress-induced mitochondrial DNA damage was reduced [111].

## Therapeutic Opportunities

Conventional therapeutic options currently available to AD patients are only able to alleviate the symptoms. These include cholinesterase inhibitors that inhibit the degradation of the neurotransmitter acetylcholine (ACh) and N-methyl-d-aspartate (NMDA) receptor antagonists (e.g., memantine) that reduce the activity of glutamate. While these options may help to slow down the degradation of cognitive functions, they do not prevent neuronal death. Hence, there is still a high morbidity and mortality rate associated with this disease [112, 113]. Nevertheless, many ongoing clinical trials are studying different treatment approaches, some of which target the aggregation of Aβ.

A growing amount of evidence is indicative of a link between telomere length, oxidative stress, and the onset and progression of AD. Hence, the discussed components involved in the maintenance and regulation of telomeres, especially telomerase, could provide a new point of focus for therapeutic strategy. Bernardes de Jesus et al. investigated the effects of telomerase gene therapy on aging. This intervention, which involved treating adult and old mice with adeno-associated virus expressing mouse TERT (AAV9-mTERT), reversed the effects of aging by reactivating telomerase. More importantly, this method of prolonging the longevity of the mice did not increase the risk of developing cancer [114].

Whittemore et al. also reported similar effects with telomerase gene therapy [58]. In this study, telomerase-deficient mice were confirmed to have reduced brain size and increased DNA damage in the hippocampus and the dentate gyrus region, and impaired neurogenesis, all of which are indicative of neurodegeneration. AAV9-TERT gene therapy was administered to these mice, and changes in several molecular biomarkers of aging were observed. In particular, there was less DNA damage and an increase in neurogenesis. A higher level of tyrosine hydroxylase, an enzyme involved in dopamine production and that is reduced in Parkinson’s disease, was also observed in the dopaminergic neurons of the mice. This study also demonstrated that an increased expression of TERT aids in ameliorating aging-related neurodegeneration-associated symptoms in both wild-type and telomerase-deficient mice models. Collectively, these results are suggestive of an effective alleviation of aging by telomerase gene therapy. However, gene therapy faces many challenges, especially in its delivery to the brain and the central nervous system, due to its low permeability through the blood–brain barrier, the complexity of the brain structure, and its route of administration [115]. Hence, numerous gene therapies that are currently undergoing different phases of clinical trials as a therapeutic strategy for neurodegenerative disease employ viral or non-viral vectors to improve their delivery and therapeuticity [116]. A study on nerve growth factor as a potential therapeutic candidate for cholinergic preservation in AD, utilizes the AAV vector, based on a successful animal model study [58, 117]. It is currently undergoing phase II clinical trial for patients with mild to moderate AD [117].

There is also an increasing amount of research on lead compounds that mimic the activity of telomerase or enhance telomerase activity [118]. For example, GV1001, a peptide vaccine derived from the active site sequence of hTERT, initially developed as an anti-cancer agent, was later found to also have anti-aging effects. In particular, it was able to relieve the effects of Aβ and oxygen–glucose deprivation/reoxygenation-mediated oxidative stress by inhibiting the production of ROS [119, 120]. GV1001 also promoted neuronal regeneration by restoring the PI3k-mTOR pathway, which is involved in cell survival and proliferation [121]. Another example would be the GSE24.2 peptide synthesized as a treatment for dyskeratosis congenita, an inherited disease characterized by premature aging and other telomerase-defective diseases [122, 123]. This peptide prevents the inhibition of telomerase and can increase the mRNA expression of TERT, thus restoring telomerase activity. GSE24.2 is also able to reduce oxidative stress by increasing the expression of antioxidant enzymes SOD1, SOD2, and catalase. The levels of 8-oxoG were reduced following the treatment of GSE24.2 [123]. Additionally, Baruch-Eliyahu et al. also reported a protective effect of AGS, a telomerase-increasing compound, against Aβ-induced neuronal degradation via the Wnt/β-catenin pathway [57]. The dysregulation of this pathway has previously been reported in several neurodegenerative diseases, including AD [124]. AGS was able to regulate the expression of certain genes and neurotrophic factors in the hippocampus of mouse models [57]. Collectively, these results provide a basis for future studies to potentially identify alternative therapeutic options for AD.

Recently, an in vitro study remodeled telomere attrition by performing a serial passaging protocol on a human hippocampal progenitor cell line. The telomere length of these cells reduced with each increasing passage, reconstituting the end replication problem. The study reported a significant decrease in the proliferation rate, without affecting the cells’ differentiation capability. Additionally, complementary RNA-sequencing analysis indicated that telomere attrition was associated with changes in over 3000 transcripts, half of which affects cognitive functions and overlaps with genes involved in neuropsychiatric disorders, namely schizophrenia and bipolar disorder [125]. Perhaps this could be looked into in future studies, with a focus on genes that are associated with AD.

## Conclusion

To date, there has been an extensive amount of research regarding Alzheimer’s. While much effort and workforce have been implemented into this area of research, the many contributing factors to the onset and progression of the disease remain to be elucidated. The amyloid cascade hypothesis suggests the overproduction and accumulation of Aβ being the initiator of a cascade of reactions resulting in AD, and there is an increasing amount of evidence indicating telomere attrition contributing to the accumulation and toxicity of Aβ. It can be concluded that changes in telomere biology, oxidative stress, underlying genetic disposition, and perceptive stress all create a positive feedback loop, resulting in the onset of AD. Further research is required to better understand the interaction between the histopathological features of the disease and the shortening of telomeres.

## Data Availability

Not applicable to this manuscript as no new dataset was generated in the study.
